# Motility Screen Identifies *Drosophila* IGF-II mRNA-Binding Protein—Zipcode-Binding Protein Acting in Oogenesis and Synaptogenesis

**DOI:** 10.1371/journal.pgen.0040036

**Published:** 2008-02-15

**Authors:** Kristin L. M Boylan, Sarah Mische, Mingang Li, Guillermo Marqués, Xavier Morin, William Chia, Thomas S Hays

**Affiliations:** 1 Department of Genetics, Cell Biology, and Development, University of Minnesota, Minneapolis, Minnesota, United States of America; 2 Department of Cell Biology, The University of Alabama at Birmingham, Alabama, United States of America; 3 Institut de Biologie du Développement de Marseille-Luminy (IBDML), CNRS UMR6216 INSERM-Université de la Méditerrannée, Marseilles, France; 4 Temasek Life Sciences Laboratory, National University of Singapore, Singapore; Washington University, United States of America

## Abstract

The localization of specific mRNAs can establish local protein gradients that generate and control the development of cellular asymmetries. While all evidence underscores the importance of the cytoskeleton in the transport and localization of RNAs, we have limited knowledge of how these events are regulated. Using a visual screen for motile proteins in a collection of GFP protein trap lines, we identified the *Drosophila* IGF-II mRNA-binding protein (Imp), an ortholog of *Xenopus* Vg1 RNA binding protein and chicken zipcode-binding protein. In *Drosophila*, Imp is part of a large, RNase-sensitive complex that is enriched in two polarized cell types, the developing oocyte and the neuron. Using time-lapse confocal microscopy, we establish that both dynein and kinesin contribute to the transport of GFP-Imp particles, and that regulation of transport in egg chambers appears to differ from that in neurons. In *Drosophila*, loss-of-function *Imp* mutations are zygotic lethal, and mutants die late as pharate adults. Imp has a function in *Drosophila* oogenesis that is not essential, as well as functions that are essential during embryogenesis and later development. Germline clones of *Imp* mutations do not block maternal mRNA localization or oocyte development, but overexpression of a specific *Imp* isoform disrupts dorsal/ventral polarity. We report here that loss-of-function *Imp* mutations, as well as *Imp* overexpression, can alter synaptic terminal growth. Our data show that Imp is transported to the neuromuscular junction, where it may modulate the translation of mRNA targets. In oocytes, where Imp function is not essential, we implicate a specific Imp domain in the establishment of dorsoventral polarity.

## Author Summary

The localization of messenger RNA is a major mechanism to generate local asymmetries in protein activities and is utilized in a diverse array of biological functions. mRNA localization and the resultant protein gradients are critical for the establishment of embryonic axes, the polarized motility of cells and neurons, and the modulation of synaptic signaling. Presently, our knowledge of the many transacting factors required for the assembly, transport, and localization of mRNAs is rudimentary. In this study, we capitalize on an *in vivo* motility assay to screen for components of actively transported RNP complexes in live *Drosophila* egg chambers. One of the components identified, *Drosophila* IGF-II mRNA binding protein or Imp, is the homolog of chicken zipcode binding protein or human IGF-II mRNA binding protein. The human IGF-II mRNA binding protein is linked to the metastatic behavior of carcinoma cells in mammary tumors, but the mechanism is unclear. We demonstrate that the *Drosophila* Imp RNP complex, is actively transported in oogenesis, as well as in neurons by the microtubule motors, dynein and kinesin. We show that the regulation of transport is distinct in oocytes and neurons and report for the first time, that *Drosophila* Imp impacts growth of the neuromuscular synaptic terminal.

## Introduction

The subcellular localization of mRNAs is a conserved means of localizing protein concentration gradients that underlie the establishment of cellular asymmetries and specialized cell functions [1,2]. For example, the localization of β-*actin* mRNA to the leading edge of neurite growth cones and embryonic fibroblasts is important for cell motility and growth [3]. Oligodendrocytes move the mRNA encoding myelin basic protein to distal processes, where it is required for myelination [4,5], and the accumulation of mRNAs in dendrites in response to synaptic activity has fostered the hypothesis that mRNA localization might mediate synaptic plasticity [6–8]. In *Drosophila* and *Xenopus* oocytes, the localization of maternal mRNA is required to establish axial polarity of the embryo [9].

The localization of mRNA requires *cis*-acting localization elements within the RNA, as well as associated *trans*-acting factors that bind the RNA and/or one another to form a ribonucleoprotein (RNP) complex [10]. Localization elements, or “zipcodes,” have been defined for a number of localized RNAs and generally reside in the UTR. The *trans*-acting proteins are required for multiple functions that include regulating mRNA translation and degradation, physically linking RNPs to the transport machinery, and tethering mRNAs to cortical anchors at specific locations [9,11]. Not surprisingly, the assemblage of *trans*-acting factors that carry out these diverse functions is complex and dynamic.

*Drosophila* oogenesis provides an excellent system in which to study the localization and transport of RNPs [12]. Germline cysts are composed of 16 cells interconnected by cytoplasmic bridges; one cell in each cyst becomes the oocyte, while the others become nurse cells that support oocyte growth. RNAs transcribed in the nurse cells are assembled into RNPs and transported to the oocyte at early stages of development, and later are correctly positioned within the oocyte. Both transport to the oocyte and localization within the oocyte are microtubule-dependent processes, suggesting directed transport of RNP complexes by microtubule motors. In early egg chambers, a polarized array of microtubules extends through the cytoplasmic bridges that connect the nurse cells with the oocyte, while later in oogenesis, microtubule reorganization facilitates proper positioning of axial determinants within the oocyte [13]. The microtubule motors, dynein and kinesin, as well as the dynactin regulatory complex, have been implicated in transport events during oogenesis, but the regulation of their interactions with specific RNP cargoes is only beginning to be addressed. Genetic screens in *Drosophila* have identified protein components of RNPs that are required for the proper localization of known mRNA axial determinants [14]. Recently, the use of live imaging techniques has allowed the direct visualization of RNP transport during *Drosophila* oogenesis [15–17] as well as apical localization of transcripts in the blastoderm embryo [18–20].

While genetic and biochemical approaches have revealed the diversity and complexity of RNPs, our understanding of their assembly, transport, localization and translational control is limited. To identify additional factors involved in these functions, we undertook a visual screen of GFP-tagged gene products that incorporate into motile particles in *Drosophila* egg chambers. Here, we report our analysis of one line with a GFP insert in the *Drosophila* ortholog of mammalian IGF-II mRNA binding protein (Imp). Imp belongs to a conserved family of proteins that regulate mRNA localization, translation and stability (reviewed in [21]. The chicken ZBP-1 and *Xenopus* Vg1-RBP were the founding members of this family, identified by their ability to bind to *cis*-acting localization elements in the 3' UTR of specific mRNAs. ZBP-1 targets the localization of β*-actin* mRNA to the leading edge of chicken fibroblasts and promotes cell migration [22]. Recent work has identified ZBP1 as a potential suppressor of the invasive behavior of mammary carcinoma cells [23]. In *Xenopus*, Vg1-RBP tethers Vg1 RNA at the vegetal pole to help establish embryonic polarity [24–26], and also regulates the asymmetric translation of β*-actin* mRNA involved in axon guidance [27–29]. In mice and humans, the related CRD-binding protein binds to and regulates the stability of mRNA, including *c-Myc* and *CD44* [30–32]. Elevated levels of Imp-related proteins in cancer cells, and their role in cell migration, have raised the level of interest in their function. Nonetheless, our knowledge of the complement of mRNAs targeted by the Imp proteins and the cytoskeletal mechanisms involved in Imp transport and localization is uncertain.

Two recent studies have provided initial characterizations of the RNA-binding functions of Imp in *Drosophila* oogenesis, with some conflicting results [33,34]. Munro et al. proposed that Imp associates with *oskar* mRNA through putative Imp-binding elements (IBE) that are required for proper localization of Imp, though not for the initial localization of *oskar* mRNA. Geng and Macdonald [34] provided evidence that Imp binds to *oskar* mRNA with low affinity, and suggested instead that *gurken* mRNA is the major target to which Imp binds [34]. They show by immunoprecipitation experiments that Imp associates with Squid and Hrp48, two RNP components with known roles in regulating *gurken* and *oskar* expression, respectively. Our work complements these analyses by examining Imp transport characteristics. We have conducted a mutational analysis of *Imp* to investigate its functions in *Drosophila*, and in addition to its redundant function in oogenesis, have identified requirements for Imp in embryogenesis and in synaptic terminal growth.

## Materials and Methods

### Protein Trap Lines and *Drosophila* Stocks

The collection of protein-trap lines is described in Morin et al. 2001 [35]. The dynein heavy chain mutations *Dhc6–6* and *Dhc6–12* are described in Gepner et al. 1996 [36], and the *khc^27^* FRT stock in Brendza et al. 2000 [37]. The *UASp-*Δ*Gl* transgenic stock, expressing a truncation of the p150/Glued subunit of dynactin, is described in Mische et al. 2007 [17]. Inducible *Imp* transgenes *UASp-RE* and *UASp-SD* were constructed using EST clones RE72930 and SD07045, respectively, which represent the two different Imp polypeptides identified in FlyBase. The *UASp-KH* transgene is a truncation derived from *UASp-RE*. Transformant fly lines were generated by standard methods [38,39]. Fly stocks for RNAi were obtained from the National Institute of Genetics, Japan, as follows: 5433R-1 (kinesin light chain) and 7765R-2 (kinesin heavy chain). All other stocks were obtained from the Bloomington Drosophila Stock Center, and are listed in FlyBase (http://www.flybase.org). We used *mata4-GAL4-VP16* (maternal a-tubulin) and *nanos-GAL4-VP16* to drive expression in the ovary; *elav-GAL4* to drive expression in the neuron; and *actin5C-GAL4* for ubiquitous expression. The molecular characterization of the *small bristle* (*sbr*) locus is described in Korey et al. 2001 [40]. The extents of duplications and deficiencies of the cytogenetic region 9E-F of the *X* chromosome, and the *stress sensitive B* (*sesB*) locus are described in Zhang et al. 1999 [41]. *Df(1)HC133* has been shown to have a breakpoint in the 5′UTR of the *Imp* gene [40]. We verified the regions included in the *Df (1) HC133* (breakpoints 9B9–10, 9E-F) and the *Y*-linked duplication, *Dp(1:Y)v^+^ y^+^* (breakpoints 9F3, 10C2; h1-h25B) by complementation.

### Inverse PCR

Inverse PCR was performed as described by the Berkeley Drosophila Genome Project. Briefly, 5μg of genomic DNA was digested with restriction enzymes *BfaI* or *HhaI*, in separate reactions. The DNA was ligated and PCR-amplified for 30 cycles using primers at the 5′ end of the P-element: *5*′*-*CTTCGGTAAGCTTCGGC-3′ (Primer 1) and 5′-CAGTGCACGTTTGCTGTTG-3′ (Primer 2); followed by reamplification of 2μl of the primary PCR product using Primer 1 and a nested primer, *5*′*-*GCACCTGCAAAAGGTCAG-3′ (Primer 3). The PCR product was ligated into the TA cloning vector pGEM-T Easy (Promega, Madison, WI), and mini-prep DNA from the resulting plasmid was sequenced using vector primers.

### P-element Excision and Breakpoint Mapping

The protein-trap transposon was excised by crossing the protein-trap flies to flies expressing Δ2–3 transposase, and selecting flies lacking the mini-*white* eye color marker carried by the P-element*.* Imprecise excisions were identified by testing white-eyed flies for *X*-linked lethality, and were maintained as balanced stocks. We examined 158 lines and identified three lethal excision events. The extents of the deletions generated by imprecise excision of the P-element were tested by complementation analyses, using deficiencies and duplications of region 9A and mutations in the flanking genes, small bristles (*sbr*) and stress sensitive B (*sesB*). One line was not rescued by a small *Y*-linked duplication of the Imp region, and probably represents a large deletion or rearrangement (data not shown). The remaining two lines, *H44* and *H149*, were rescued by the *Y*-linked duplication, and failed to complement the deficiency. Deletions were characterized molecularly by blot analysis and by sequencing targeted regions of genomic DNA.

### Germline Clone Analysis

To assess the requirement for Imp during oogenesis, lethal excision lines were recombined onto chromosomes containing FRT inserts at 14A-B (genotype: *y w v P[mini-w+,FRT]101*). Recombinant chromosomes were identified using the marker *yellow* and the mini-*white* marker associated with the FRT insertion. Presence of the excision mutations was determined by testing the recombinant chromosomes for *X*-linked lethality. Germline clones were produced in the presence of the dominant female sterile mutation, *ovo^D1^*, by crossing balanced excision-FRT females to males of the genotype *w, ovo^D1^, v P[mini-w+,FRT]101; P[hsFLP]38*. Eggs were collected for 3–4 days and then larvae were heat-shocked for 1.5 hours in a 37°C water bath to induce expression of the FLP recombinase enzyme. Females of the genotype *excision-FRT/ovo^D1^-FRT; hsFLP* were crossed to sibling males and then examined for the presence of eggs and larvae, or ovaries were dissected and fixed for antibody staining. The lethal phase analyses were conducted by standard protocols and similar to those previously described [42].

### Protein Methods

Sucrose density gradient analysis was based on Wilhelm et al. 2000 [43]. Extracts were prepared from hand-dissected ovaries in DXB (25mM Hepes, pH 6.8, 50mM KCl, 1mM MgCl2, 1 mM DTT, 250 mM sucrose) plus protease inhibitors (10 mg/ml aprotinin, 1 mg/ml leupeptin and pepstatin, 0.1 mg/ml each of soybean trypsin inhibitor, *n*-tosyl L-arginine methylester, and benzamidine). Samples were treated with RNasin ribonuclease inhibitor (Promega), or, in parallel, RNase A followed by RNasin. Extracts were clarified at 100,000 × *g* prior to loading 700 μg total protein on 5 ml 10–40% sucrose gradients prepared in DXB, and centrifuged for 5 hrs at 44,000 rpm in a Beckman SW 50.1 rotor. Gradients were collected into 250μl fractions and frozen at −80°C. 15 μl of each fraction were analyzed by immunoblot.

To examine protein expression levels, hand-dissected ovaries were homogenized and spun for 15 minutes at 4°C in a microfuge. Equal amounts of total protein were separated by SDS-PAGE and transferred to PVDF membrane by standard methods. Blots were processed using the Tropix chemiluminescence system (Applied Biosystems). Anti-GFP monoclonal JL-8 (BD Biosciences) was diluted 1:1000. The anti-Imp antibody was a gift from Paul Macdonald [34].

### Microscopy

#### Confocal microscopy of living tissue.

Ovaries were dissected from protein-trap females directly into halocarbon oil on 24mm × 50mm coverslips. Individual ovarioles were teased apart with a 27-gauge needle and separated from the overlying sheath by gently pulling the ovarioles across the coverslip. Images were acquired using a Nikon Eclipse TE200 inverted microscope equipped with a PerkinElmer Confocal Imaging System (PerkinElmer, Inc), and Hamamatsu's Orca-ER digital camera. GFP-Imp particle movements in nurse cells were captured at one-second intervals and 2x2 binning using a 60× planapo (NA 1.4) objective. GFP-Imp localization was imaged with 1x1 binning using a 40× planapo (NA 1.3) objective and 1μm optical sections. To visualize axonal transport, wandering third instar larvae were pinned down to a sylgard dish and dissected dorsally in HL3 buffer [44,45]. The gut and fat body were removed to expose the nerves and GFP-Imp was imaged at 1 second intervals with 2x2 binning and a 100× oil planapo (NA 1.4) objective. To drive RNAi in segmental axons by *elav-GAL4*, crosses were set at 25°C, and resulting first instar larvae were shifted to 29°C; then third instar larvae were examined as described above.

#### Larval and adult motility.

The behavior of larvae, as well as adult flies were compared under a Zeiss stereoscope. Videos of both the mutant and sibling wild type animals were made using Nikon camcorder.

### Immunohistochemistry

Ovaries were dissected from 2–4 day old females and fixed in formaldehyde/heptane as described in [46]. Fixed ovaries were examined for GFP expression or stained with antibodies diluted as follows: dynein heavy chain (P1H4) 1:500 [46], kinesin heavy chain 1:1500 (Cytoskeleton), Gurken 1:10 (T. Schupbach, Princeton University), Staufen 1:3,000 (D. St Johnston, University of Cambridge), BicD 1:10 (R. Steward, Rutgers University; clones 4C2 and IB11), Oskar 1:100 (A. Ephrussi, EMBL Heidelberg), and Orb 1:25 (Developmental Studies Hybridoma Bank at University of Iowa). Egg chambers were examined by confocal microscopy, on either a Nikon TE200 or a Zeiss Axiovert 200M microscope. To examine synaptic morphology, third instar larvae were dissected in PBS, fixed in 4% formaldehyde for 20 minutes, then washed and processed as for ovaries. The anti-cysteine string protein (CSP) antibody 6D6 (Developmental Studies Hybridoma Bank at University of Iowa) was diluted1:200, and anti-dFMR1 antibody 5A11 (Developmental Studies Hybridoma Bank at University of Iowa) was diluted 1:1000. Rabbit anti-phosphorylated Mad (PS1) was diluted 1:500 [47]. Alexa-488 or −567-conjugated secondary antibodies (Molecular Probes) were used at final concentration 1:200.

### Cytoskeletal Disruption

For colcemid treatment, young females were starved for 3 hours prior to treatment. Flies were fed a paste of baker's yeast made with either 200μg/ml demecolcine (colcemid) (Sigma D7385) or, as a control, 200μg/ml lumicolchicine (Sigma L0760), and were dissected after 24 hours of feeding. Ovaries were fixed as above. For cytochalasin D treatment, ovaries were dissected from 2–4 day old females into 1X Robb's Minimal Medium with or without 20μg/ml cytochalasin D. Individual stage 8 and 9 egg chambers were teased apart and incubated 10–20 minutes at room temperature before imaging.

### Statistical Analyses

GFP particle velocity and run-length were manually tracked using the “track points” function of Metamorph (Molecular Devices) image analysis software. Particles displaying linear movement for four consecutive frames were selected for analysis. The cursor was placed at the leading edge of each particle, and the X and Y positions were recorded. As the particle moved in each subsequent frame, the cursor was moved to the new position of the leading edge, and the new X and Y positions were recorded. This procedure continued until the particle ceased moving, or moved out of the plane of focus; consequently, the measurements of run lengths are underestimates. Mean velocity and run-length, and standard deviation, were calculated for each particle.

To analyze synaptic terminal size, the NMJ at muscles 6 and 7 of abdominal segments 2 and 3 were examined to determine the number of synaptic boutons and muscle size. For each genotype, the synaptic terminal size was calculated as the total number of synaptic boutons in each hemi-segment divided by the surface area of the respective muscle. This relative synaptic terminal size was then averaged for each genotype.

Statistical significance of the difference between mutant and wild type was determined using Student's t-test. Significance was established when *p* < 0.05.

## Results

### A Visual Motility Screen Recovers RNP Components

To identify molecules that are actively transported in oogenesis, we screened a previously isolated collection of *Drosophila* GFP-protein trap lines [35]. These transgenic lines were generated using a protein trap transposon (PTT) designed to tag proteins with GFP by random insertion within introns, generating an artificial GFP-exon that is spliced into mRNAs. The recovered lines were originally screened for GFP expression during embryonic and larval development [35]. We rescreened the collection to identify motile GFP particles in the nurse cells and oocytes of developing egg chambers. Ovaries were dissected live into halocarbon oil and individual egg chambers were examined for the pattern of GFP expression. Lines that showed GFP enrichment in the oocyte relative to the nurse cell cytoplasm were subsequently examined by time-lapse confocal microscopy for GFP particle movement, and were characterized molecularly to determine the identity of the trapped genes.

Using this strategy we screened more than 300 trap lines and identified several genes, including the known RNP components, Ypsilon schachtel (Yps) and *Squid* (Videos S1 and S2). Yps has been found in an RNP complex with Exuperantia, and is reported to antagonize Orb function in the localization of *oskar* mRNA [43]. Squid also assembles into RNP complexes and is required for the proper localization of the dorsoventral determinant, *gurken* mRNA [48]. Our live imaging shows the GFP-tagged Yps and Squid gene products each incorporate into morphologically distinct particles, which move with significantly different velocities (Yps: 0.59 μm/s ± 0.16 s.d., compared to Squid: 0.50 μm/s ± 0.10; *p* < 0.05) and different average run lengths (Yps: 4.28 μm ± 1.74, compared to Squid: 3.89 μm ± 1.64; *p* < 0.05). The differences in motility parameters for each of the GFP-tagged proteins suggest they incorporate into distinct particles subject to distinct regulatory mechanisms.

**Figure 1:**
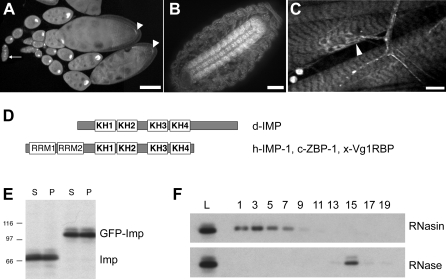
GFP-Imp Is Expressed in Ovaries and Neurons (A) In the ovary, GFP-Imp is detected within the germarium as early as stage 1 (arrow) and accumulates in the presumptive oocyte in early egg chambers. By stage 9–10, GFP-Imp is concentrated at the oocyte posterior pole (arrowheads). Bar, 25 μm. (B) GFP-Imp is enriched in the embryonic central nervous system (Bar, 25μm), and (C) in larval segmental axons and at the synapse (arrowhead). Bar, 5μm. (D) Schematic highlights the domain structure of *Drosophila* Imp compared to orthologs in human, chick, and *Xenopus*. RRM = RNA recognition motif. KH = hnRNP K homology domain. (E) The insertion of the PTT does not alter the level of Imp protein expression. Ovary extracts from wild type and GFP-Imp flies show similar levels of overall expression, as well as similar relative expression in supernatant (S) and pellet (P) fractions, as detected by an anti-Imp antibody. (F) GFP-Imp is present in a large, RNase-sensitive complex. GFP-Imp ovary extracts were fractionated over a 10%–40% sucrose gradient in the presence of RNase, or RNase inhibitors. Equal volumes of alternate fractions were analyzed by western blot using anti-GFP antibody. Lane L, sample of extract loaded on gradient. Fraction numbers are indicated above the lanes (bottom of gradient is fraction 1).

We also identified a single PTT insertion within a large intron of *Imp*, an X-linked gene encoding the *Drosophila* ortholog of human IGF-II mRNA binding protein. The GFP-Imp particle exhibits robust motility in the nurse cells (Video S3) and shows distinct accumulation in the oocyte, as well as in the embryonic and larval central nervous system (Figure 1A–1C). Imp is a member of a conserved family of zipcode-binding proteins which regulate the localization, translation and stability of multiple mRNAs [21]. Imp is about 47% identical in sequence to its vertebrate orthologs across the region of four KH-type putative RNA-binding domains [49]. Two RNA recognition motif (RRM) domains are absent from *Drosophila* Imp, which is shorter at the N-terminus and longer at the C-terminus than the vertebrate proteins [49] (see [48], and Figure 1D). The level of Imp protein expression is apparently unaffected by the GFP insertion (Figure 1E), and flies are homozygous viable and fertile, suggesting that the tag does not impair Imp function.

To address whether the GFP-Imp particle is a RNP, we examined its stability following treatment with RNase. Extracts from GFP-Imp ovaries were fractionated over 10–40% sucrose gradients in the presence of RNase inhibitor or after treatment with RNase A (Figure 1F). Intact complexes migrate near the bottom of the gradient, with sedimentation coefficients greater than 20S. Treatment of cell extracts with RNase A disrupts the complex, shifting the GFP-Imp peak toward the top of the gradient. These results indicate that GFP-Imp is part of a large RNA-containing complex in *Drosophila*, and are in agreement with recent reports describing the interaction of Imp with maternal and neuronal RNAs [33,34,50].

### Dynein Is Required for Imp Particle Motility in Egg Chambers and in Axons

**Figure 2:**
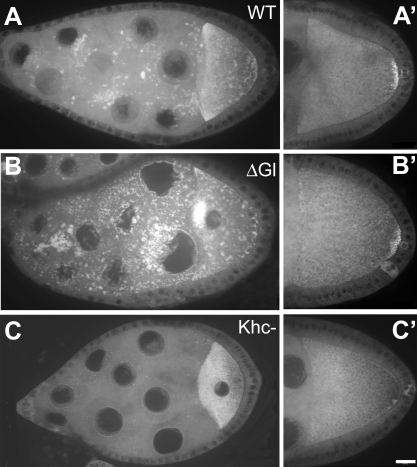
GFP-Imp Localization in the Oocyte Requires Microtubule Motor Function (A, A') GFP-Imp expression in wild type egg chambers is enriched in the oocyte and localizes to the posterior of the oocyte in stage 10. (B, B') Disruption of dynein using ΔGl overexpression (or the *Dhc 6–6/6–12* transheterozygous mutant background; data not shown) produces an early aberrant accumulation of GFP-Imp to the oocyte anterior. However, the subsequent accumulation to the posterior pole is not affected. (C, C') In germline clones homozygous for the kinesin null mutation, *khc^27^*, GFP-Imp enrichment in the early oocyte is retained, but later accumulation at the posterior of the oocyte is lost. Bar (applies to all images), 10 μm.

During oogenesis, GFP-Imp RNP particles are rapidly transported through the nurse cell compartment, become enriched in the early oocyte, and then later are concentrated at the posterior pole of the oocyte (Figures 1 and 2). We used time-lapse confocal imaging to document the unidirectional, linear movements of GFP-Imp particles in nurse cells (Video S3). Similar to other maternal RNP particles, the transit of GFP-Imp from nurse cells to the oocyte requires microtubules, but not actin filaments [17,51–53]. Movement of GFP-Imp to the oocyte is completely blocked in egg chambers collected from females treated with the microtubule-depolymerizing drug colcemid (Figure S1). In contrast, the actin inhibitor, cytochalasin D, does not block GFP-Imp motility in nurse cells (Table 1), but does disrupt its retention at the posterior pole of the oocyte (Figure S1).

**Table 1:**
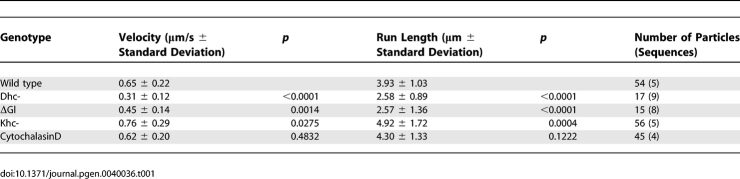
Transport of GFP-Imp in Nurse Cells

**Figure 3:**
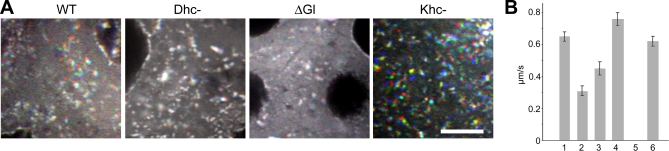
Dynein and Kinesin Have Opposing Effects on GFP-Imp Motility in Nurse Cells (A) Three sequential images are separately pseudocolored red, green, or blue, and overlaid to highlight the frequency of GFP-Imp particle movements in the nurse cell cytoplasm in wild type and mutant backgrounds. Motility is easily discerned in wild type by the resolution of individual colors. After disruption of dynein (Dhc-*)* or dynactin (ΔGl), there is almost no movement; particles appear white, resulting from the superimposition of red, green, and blue. In contrast, disruption of kinesin I (Khc-) increases particle transport, as demonstrated by the increased visibility of individual colors. Bar, 10 μm. Genetic backgrounds shown include Dhc-, [*Dhc6–6/Dhc6–12*]; Δ*Gl*, [*UASp-*Δ*Gl/+; nanos-GAL4/+*]; and khc^27^, [*khc^27^*/ *khc^27^* ] germline clones, as described in the text. (B) Motor mutations affect velocity as well as frequency of GFP-Imp transport events. Histogram illustrates the average velocities of GFP-Imp particles in nurse cells derived from wild type and mutant backgrounds. (1) wild type, (2) *Dhc6–6/Dhc6–12*, (3) *UASp-*Δ*Gl/+; nanos-GAL4*, (4) *khc^27^*/ *khc^27^*germline clone, (5) wild type treated with colcemid, (6) wild type treated with cytochalasin D.

We analyzed particle movements in genetic backgrounds with reduced dynein and kinesin function, to identify the motor responsible for Imp transport to the oocyte (Table 1; Figure 3). In transheterozygous combination, the hypomorphic dynein heavy chain alleles *Dhc6–6* and *Dhc6–12* produce female sterile adults [36]. Egg chambers in these females arrest in late oogenesis, and normal dynein localization in the oocyte is disrupted [46]. Although particle movement is not completely abolished in the dynein hypomorphic mutant egg chambers, the number of GFP-Imp particles in motion is substantially reduced compared to wild type. In addition, the average particle velocity decreases from 0.65 μm/s ± 0.22 in wild type to 0.31 μm/s ± 0.12 in the dynein mutant. As another way to test dynein's contribution to GFP-Imp particle motility, we disrupted the function of dynactin, the dynein activating complex. We used *nanos-GAL4* to drive germline expression of a dominant negative dynactin construct, *UASp-*Δ*Gl* [17] and observed a reduction in transport velocities within the nurse cells (0.45 μm/s ± 0.14), and the early mislocalization of GFP-Imp to the anterior of the oocyte (Figure 2B). As in the dynein mutant, the subsequent accumulation of GFP-Imp at the posterior pole of the oocyte still occurs (Figure 2B'). We examined the role of kinesin in GFP-Imp transport using germline clones homozygous for a null mutation in the kinesin heavy chain, *khc^27^* [37]. In contrast to the dynein mutant background, particle velocity increases when kinesin function is lost (0.76 μm/s ± 0.29**)**. In the kinesin mutant background, the posterior localization of GFP-Imp is blocked (Figure 2C and 2C'), similar to the behavior of the posterior determinants *oskar* mRNA and Staufen protein [17,54].

GFP-Imp localizes to the developing nervous system during embryogenesis and also during late larval development (Figure 1B and 1C), where it exhibits rapid transport in the segmental axons (Video S4). Intriguingly, GFP-Imp particle movement along axonal microtubules is saltatory and bidirectional, exhibiting abrupt, short and frequent reversals in the direction of transport along a microtubule, regardless of whether transport is anterograde or retrograde. The reversals in GFP-Imp motility along axonal microtubules are not observed in the motility of GFP-Imp particles in the egg chamber, suggesting that transport is differentially regulated in the different cell types. The velocities of GFP-Imp particles ranged from 0.41 μm/s to 2.13 μm/s; we did not separately establish the minus- and plus-end velocities due to the mixed polarity of microtubules. As in ovaries, motility of GFP-Imp in axons requires microtubule motors. To reduce dynein motor activity, we expressed *UASp-*Δ*Gl* with *elav-GAL4* and observed a significant decrease in particle velocities (*p* < 0.0001; Table 2). To disrupt kinesin I function, we used dsRNAi to knock down the kinesin heavy and light chain polypeptides, again using the *elav-GAL4* driver. Unlike ovaries, loss of kinesin function in the axon has a similar effect on Imp motility as loss of dynein, resulting in a significant decrease in velocity (*p* < 0.0001). The antagonistic relationship apparent between the dynein and kinesin motors in ovaries is not present in axons (Table 2).

**Table 2:**
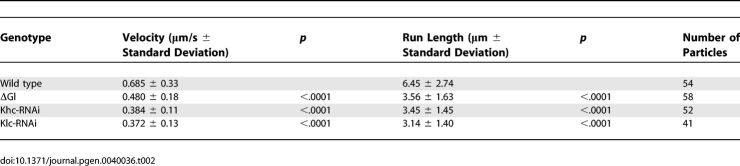
Transport of GFP-Imp in Segmental Axons

### Differential Function of Imp Splice Variants

We generated mutations in the *Imp* gene by imprecise excision of the PTT, and identified two excision lines, *H44* and *H149*, which are homozygous lethal. These lines fail to complement a deficiency, *Df(1)HC133*, that has been shown to remove the entire *Imp* coding sequence [40], and their lethality is rescued by a small Y-linked duplication of the *Imp* region (Table 3). Additionally, both lines complement mutations in the two flanking genes, *small bristles* (*sbr*) and *stress-sensitive B* (*sesB*)**.** These results provide genetic evidence that the mutations affect only *Imp*, and demonstrate that Imp function is essential for *Drosophila* development. Molecular analyses of genomic DNA from each mutant line confirm that both alleles disrupt *Imp*, but not the adjacent genes. *H149* contains an internal ∼5.1 kb deletion, which removes the intron containing the PTT element, as well as several adjacent exons. In the case of the *H44* allele, an inversion and rearrangement of the PTT disrupts *Imp* exons. Consistent with these results, we do not detect Imp protein in extracts derived from mutant ovaries (Figure S2).

**Table 3:**
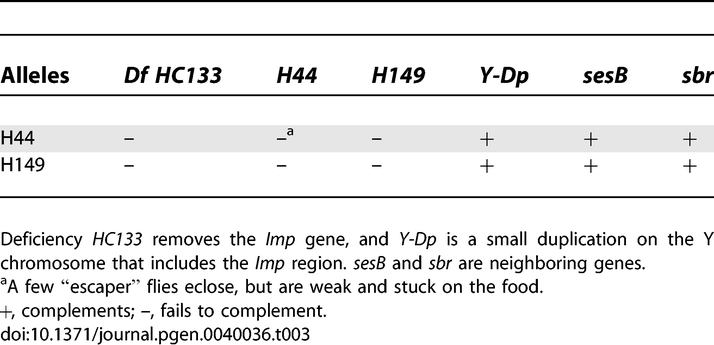
Complementation Analysis of *Imp* Alleles *H44* and *H149*

To examine whether Imp is involved in the establishment of oocyte polarity, we generated germline clones homozygous for the lethal *Imp* mutations. We find that females with germline clones for either the *H149* or *H44* mutation produce phenotypically normal eggs. The expression and localization of known determinants of the anterior/posterior axis (Staufen, Bicaudal-D, Orb, and Oskar) and dorsal/ventral axis (Gurken) appear normal in the clonal egg chambers (data not shown). Although no visible defects were observed in germline clones for either *Imp* mutation, the hatching rates of the resulting embryos were reduced. For *H44*, 84.5% of the embryos failed to hatch (*n* = 328), compared to 8.2% for wild type (*n* = 348). Cuticle preparations from *H44* dead eggs displayed two phenotypes. The first category of embryos looked normally developed, even though they still failed to hatch. The second category of embryos arrested earlier in development and appeared to have defects in the anterior of the embryo, perhaps in either germband retraction or head involution. All the surviving adult progeny that arose from germline clones were female, suggesting zygotic rescue by the paternal contribution of a wild type *X* chromosome. By comparison, Munro et al. (2006) previously reported 100% of the embryos derived from *Imp* mutant clones died in late embryogenesis [33]. As a more sensitive assay of Imp function in germline development, we examined the adult progeny produced from the homozygous mutant egg chambers to test for a “grandchildless” phenotype. These female progeny were fertile, supporting the conclusion that maternal Imp function is not required for the production of a normal female germline.

To further probe the role of Imp in oogenesis, we overexpressed three engineered transgenic constructs. The *Imp* gene is predicted to have multiple transcripts that encode two different polypeptides varying in their N-termini, but sharing identical C-terminal KH-domains. Our *Imp* cDNA transgenes, *UAS-RE* and *UAS-SD*, encode these two distinct polypeptides. A third transgene, *UAS-KH*, is a truncation of *Imp* that encodes only the four KH domains and lacks the N-terminal sequences (Figure S2A). Germline expression of all three constructs was driven with maternal *alpha-tubulin-GAL4* (*a-tubGAL4*). Flies expressing the *UAS-SD* transgene (*a-tubGAL4/+; UAS-SD/+*) exhibit a dorsal appendage phenotype (Figure S2C) and failed to hatch. Multiple independent transgenic lines were tested and all lines produced a similar dorsal appendage defect suggesting the phenotype is specific to the expression of the *UAS-SD* transgene. The penetrance of the phenotype varied between lines from 29% to 47% (for each line, *n* > 270), consistent with variation in the levels of expression of the transgene in the different transgenic lines. A similar defect in dorsoventral polarity was reported by Geng *et al*. (2006) [34]. However, flies overexpressing either the *UAS-RE* (*a-tubGAL4/+; UAS-RE*) or the *UAS-KH* (*a-tubGAL4/+; UAS-KH/+*) transgene, show no deffect in oocyte development or female fertility (Table 4). These results identify an N-terminal region preceding the KH domains that is important for directing Imp function in oogenesis.

**Table 4:**
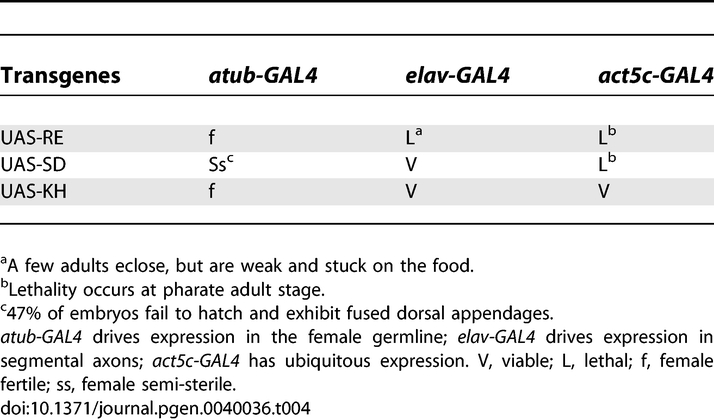
Tissue-Specific Overexpression of Three *Imp* cDNAs

### Imp Functions in Synaptic Terminal Growth

**Figure 4:**
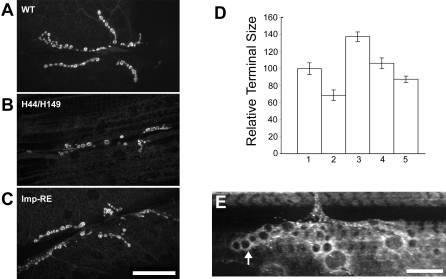
Imp Expression Affects Synaptic Terminal Size at the Neuromuscular Junction (A) Individual synaptic boutons are clearly labeled by the anti-CSP antibody. Wild type larval synapse is shown. (B) Bouton numbers are decreased in the *Imp* mutant combination, *H44/H149*. (C) Presynaptic overexpression of the *UAS-RE* transgene by *elav-GAL4* (*elav-GAL4*/+; *UAS-RE*/+) significantly increases the number of boutons. Bar (applies to A–C), 50 μm. (D) Histogram compares relative synaptic terminal sizes of (1) wild type, (2) *Imp H44/H149* mutant combination, and (3–5) overexpression of *Imp* transgenes, including (3) *elav-GAL4/+; Imp-RE*, (4) *elav-GAL4/+; Imp-SD/+*, and (5) *elav-GAL4/+; Imp-KH/+*. The synaptic terminal size was defined as the number of synaptic boutons normalized to the surface area of muscle 6/7, and the reference was set to 100 for the wild type control. (E) GFP-Imp is present at the synapse and accumulates along the membrane cortex of synaptic boutons (arrow). Bar, 5 μm.

The loss-of-function *Imp* alleles, *H44* and *H149*, exhibit increased lethality late in pupal development, at the pharate adult stage. Pharate adult lethality is commonly observed for mutants defective in synaptic transmission, and is consistent with a role for Imp in mRNA localization and translation during synaptogenesis [55–58]. We examined the neuromuscular junction (NMJ) in third instar larvae from homozygous and transheterozygous (*H44*/*H149*) *Imp* mutant backgrounds, and counted the numbers of synaptic boutons (axon terminal structures). Since the size of the synaptic terminal grows dramatically during the third larval instar stage, we took care to select larvae that were of comparable size for our analysis. Additionally, to correct for any size differences, we calculated and compared the relative terminal size for all lines (see Materials and Methods). From this analysis, we observed significantly smaller synaptic junctions in the loss-of-function mutant backgrounds (e. g. *H44*/*H149* larvae, *p* < 0.01; Figure 4). The reduction in size of the synaptic termini did not result in any obvious muscle twitching in mutant larvae, and although we did note that mutant larvae appear somewhat sluggish, it is difficult to quantitate (compare Videos S5 and S6). To further assess a neuromuscular defect, we examined the behavior of the adult mutant flies that escaped the pharate adult lethal phase and eclosed. These “escaper” flies have a largely normal morphological appearance, with the exception that their wings are raised and held back. The mutant flies are not able to climb the walls of the vials, but remain on the food and exhibit little movement. Closer inspection shows that the movement of the mutant flies is severely compromised, with highly uncoordinated twitching and grooming leg movements. The mutant flies frequently fall over as they try to walk and cannot readily upright themselves (Videos S7 and S8). These phenotypes are consistent with a neuromuscular defect, but in this transheterozygous mutant background the loss of Imp function extends beyond the nervous system. The apparent neuromuscular phenotype may result from the indirect consequences of defects in other tissues. We tested this possibility by examining animals (*elav-GAL4/+; UAS-RE)* that overexpress the *UAS-RE Imp* transgene presynaptically in neurons using *elav-GAL4*. Similar to H44/H149 mutant larvae, the motility of larvae expressing the UAS-RE Imp transgene in neurons appeared sluggish (Videos S9 and S10). Only 4% of the expected test class eclosed, and the surviving adults have locomotion defects that are very similar to the transheterzygous (H44/H149) Imp mutant flies. The flies exhibit sporadic tremors in the legs, an unstable walk, and frequently fall over and do not easily get back up (Videos S11 and S12). Surprisingly, *UAS-RE* overexpression generated a significantly larger synaptic terminal (*p* < 0.0001). Thus elevated levels of Imp expression and larger synaptic termini also appear to disrupt proper neuromuscular function. In parallel experiments, the overexpression of either *UAS-SD* or *UAS-KH,* using *elav-GAL4* had no effect on survival, adult eclosure or synaptic terminal size (*p* > 0.05). In contrast to its apparently redundant function during oogenesis, Imp is required presynaptically for NMJ growth and may regulate neuromuscular activity.

**Figure 5:**
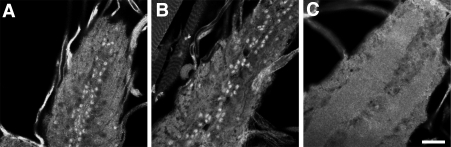
*Imp* Mutations Do Not Disrupt the Nuclear Accumulation of pMad (A) In wild type, pMad is readily detected in the nuclei of motorneuron ganglia. (B) Nuclear uptake of pMad is retained in the *Imp* transheterozygous *H44/H149* mutant background. (C) In contrast, pMad staining is abolished in the *wit* mutant. Bar, 10 μm.

Genetic analysis has shown that the Bone Morphogenetic Protein type II receptor, Wishful thinking (Wit), is also required for proper growth of the *Drosophila* NMJ, and similar to Imp exhibits a lethal phase during late pupal development [56,59]. The Wit receptor is required presynaptically, where it is thought to transduce a retrograde signal from the synapse to the cell body to coordinate synaptic growth with muscle growth. Wit signaling results in the phosphorylation and nuclear accumulation of the transcription factor, Mothers against dpp (Mad). We hypothesized that Imp might regulate the translation of synaptic RNAs that encode components of this retrograde signaling pathway. Consistent with this possibility, GFP-Imp is detected in the synaptic boutons (Figure 4E). If Imp mediates Wit signaling, then the loss of Imp function, which reduces terminal size, would be predicted to diminish Wit signaling. To test this hypothesis we asked if the nuclear accumulation of phospho-Mad (pMad) is blocked in the *Imp* mutant backgrounds (Figure 5). Unlike the requirement for Wit function, our results show that Imp function is not necessary for the accumulation of endogenous pMad in the larval ventral ganglion. We conclude that the requirement for Imp is downstream of Wit and the nuclear action of pMad. Alternatively, Imp function may lie in a separate, parallel pathway that regulates synaptic terminal growth.

## Discussion

In a visual screen for gene products that are actively transported in oogenesis, we recovered Imp, the *Drosophila* ortholog of the chicken zipcode binding protein, ZBP-1. We confirm recent reports that while Imp is essential, it is not required for oocyte development or the proper localization of maternal determinants. However, results of our overexpression experiments are consistent with a redundant role for Imp in dorsoventral patterning, as previously reported [34]. From our study of Imp function in neurons, we show that Imp is required for the proper growth and/or maintenance of the synapse at the NMJ.

Our analysis of the GFP-Imp trap line extends previous studies to characterize transport of Imp in both ovaries and axons. We show that Imp motility requires microtubule motor function. Similar to Exuperantia and Staufen RNPs [17], the number and velocity of GFP-Imp particles in nurse cells is significantly reduced in a hypomorphic dynein mutant background. Loss of dynein function lowers the velocities of all Imp particles, suggesting that dynein is the motor that actively transports Imp in the nurse cell compartment. Moreover, we find that particle velocity is elevated in the kinesin null background, as we previously found for Stau-GFP and Exu-GFP RNP transport. This antagonistic interaction of dynein and kinesin suggests that both motors reside on the Imp RNP complex, but that in nurse cells only dynein actively promotes RNP translocation along microtubules [17]. Consistent with this interpretation, we do not observe saltatory, bidirectional movement of the Imp particles along microtubules in nurse cells. In contrast, Imp transport along microtubules within larval axons is saltatory, with frequent, short reversals in direction of transport. In axons, disruption of either dynein or kinesin reduces the velocity of dImp transport. It will be important to identify signaling pathways which regulate plus- and minus-end motor activities and consequently determine the directional bias of RNP transport in different tissues.

The transport and localization of ZBP-1 particles at the leading edge of chicken fibroblasts is predominantly an actin-based process; localization of β-actin mRNA and ZBP-1 are disrupted by treatment with cytochalasin D, but not colchicine [60]. Moreover, chemical inhibition of myosin ATPase activity blocks ZBP-1 accumulation at the leading edge of fibroblasts [61]. We have no evidence that actin mediates active transport of *Drosophila* Imp, but the retention of Imp at the posterior pole of the oocyte is cytochalasin-sensitive and may require actin. Although *Drosophila* Imp lacks the RNA-binding “RRM” domains present in vertebrate orthologs, it contains the four KH domains which are reportedly sufficient for ZBP-1 association with actin filaments, as well as for RNP granule formation [22]. Nielsen et al. (2002) showed the KH domains of human Imp1 were sufficient for its correct localization and association with microtubules [62]. How the KH domain can mediate association with both actin and microtubules is not clear.

The transport and localization of GFP-Imp during *Drosophila* oogenesis suggested a role for Imp in the localization of maternal RNA determinants that specify the embryonic axes. However, in germline clones of Imp loss-of-function mutations, we and others find no evidence of mispositioned mRNA determinants for either the anterior/posterior or dorsal/ventral axes [33,34]. We also looked at expression of Oskar protein in Imp germline clones and found no evidence that Imp is required for translational repression of oskar mRNA. This could reflect the existence of a functionally redundant factor that compensates for the loss of Imp during oogenesis [33].

Geng and MacDonald (2006) report that reducing Imp function partially suppresses a gurken mRNA misexpression phenotype [34]. In addition, Imp overexpression disrupts gurken mRNA localization and alters dorsoventral polarity, as reflected by defects in dorsal appendage formation. We observe a similarly penetrant dorsalization of the eggshell upon overexpression of the Imp splice variant encoded by our transgene, UAS-SD. This isoform varies from the alternate Imp polypeptide only in a small N-terminal region preceding the four KH domains. According to a recent analysis, the polypeptide represented by UAS-SD is normally expressed within the germline [63]. The isoform encoded by UAS-RE is apparently not abundantly expressed in the germline, and we find that its ectopic expression there does not generate the dorsalized phenotype. We propose that a domain preceding the KH domains and unique to the isoform represented by UAS-SD mediates an interaction that is critical for the proper regulation of Imp function in the germline.

Our observations also address the zygotic function of Imp and show that Imp is essential for embryonic development. In contrast to the lack of germline phenotypes in oogenesis, a significant proportion of embryos derived from homozygous Imp mutant eggs fail to hatch. This phenotype appears unrelated to the function of maternal determinants, and as suggested by Munro and colleagues (2006) may result from the misregulation of other IBE-containing transcripts in the absence of Imp [33]. RNA targets are known to regulate cell migrations that contribute to tissue morphogenesis in other organisms [27,64]. Similarly, the loss of *Drosophila* Imp function might disrupt cell migration in early embryonic development. We noted anterior defects in cuticle preparations that could result from such defects. Imp is also required late in development; strong loss-of-function mutants die as pharate adults. The late lethal phase is consistent with neuronal function and the observed enrichment of Imp in the central nervous system (Figure 1B) [65]. Strong mutations in *Drosophila* dFmr1 also die as pharate adults [66]. dFMR1 is another KH-type RNA binding protein and overexpression or loss-of-function dFmr1 mutations are reported to generate neuronal and behavioral phenotypes [67,68]. Consistent with the similar phenotype, dFMR1 associates with Imp in neuronal RNP granules [50]. We report here that dFMR1 also associates with Imp in *Drosophila* ovaries, suggesting that the function of a dFMR1/Imp complex in RNA localization and translational control is not specific to neurons (Figure S3). The distribution of Imp and dFMR1 in neurons overlaps but is not identical, consistent with the interpretation that the two RNP components are not obligate partners. An earlier study has suggested that a fraction of the dFMR1 pool associates with PAR and lethal giant larvae proteins in a RNP complex [69]. How broadly Imp distributes among different RNPs is not known.

Previous studies have reported that the overexpression of *Drosophila* Imp in the larval nervous system generates defects in axonal pathfinding and neural development [70,71]. We extend the analysis of Imp function in neurons and provide evidence that Imp acts to modulate synaptic terminal growth. We observe a decrease in the size of the synaptic terminal at the NMJ in loss-of function mutant backgrounds, and detect an expansion of the terminal when the UAS-RE transgene is overexpressed in neurons. The alterations in the size of synaptic termini correlate with aberrant neuromuscular activity. The defects in larval motility are mild, while eclosed adult flies exhibit severe neuromuscular defects, most notably very limited and uncoordinated sporadic leg movements. The defects observed are similar in both the loss-of-function Imp mutant background and in flies in which the RE isoform of Imp is specifically overexpressed in neurons. These results suggest that aberrant levels of Imp in neurons, either high or low, and the resultant increased or decreased size of synaptic termini can disrupt neuromuscular activity. Our results suggest that Imp function in neurons is not restricted to the guidance of growth cones during embryogenesis, and we speculate that Imp is also involved in the active delivery and translation control of transcripts at synaptic termini. The localization of Imp in synaptic boutons is not homogenously distributed, but appears to be enriched along the membrane. Thus, similar to the *Drosophila* oocyte, RNPs and the associated functional mRNA determinants become tethered to the cortical domains where local translation can quickly respond to changes in synaptic activities. Intriguingly, the delivery and local translation of mRNAs may regulate synaptic plasticity and homeostasis. However, we have not directly characterized the electrophysiological activity of synaptic terminals and can only speculate that the observed defects in neuromuscular activity result from the aberrant sizes and transmission of synapses in the Imp mutants. Moreover, it is worth noting that the electrophysiological activity and synaptic terminal size at the NMJ are not always tightly coupled. For example, highwire mutants have synaptic terminals that are double the size of controls, but the quantal content is reduced [72]. Similarly, spin mutants have a small or normal quantal content but a substantially increased synaptic terminal [57,73]. The opposite situation is also possible; presynaptic rescue of gbb mutants results in normal quantal content with a small synaptic terminal size [74]. All mutants in the BMP signaling pathway in *Drosophila* have a similar phenotype of decreased synaptic terminal size and quantal content [56,59,74–76]. In wit mutants the synaptic terminal at the NMJ is 60% of normal size, while quantal content is about 20% of controls [56,75]. Despite these significant defects at the morphological and electrophysiological level, wit larva do not show any obvious locomotion defects.

A critical set of questions concerns how the transport and anchoring of Imp RNP complexes are influenced by signaling pathways. TGF-ß Bone Morphogenetic Protein signaling is required for synaptic development and plasticity in *Drosophila*. Mutations in either the ligand, glass bottom boat, or the corresponding type II receptor, Wit, impair synaptic growth and have a similar late lethal phase. The downstream consequence of Wit signaling is the nuclear accumulation of pMad. Our results establish that nuclear accumulation of pMad is not disrupted by the loss of Imp function and suggest that the mRNA targets of Imp do not function upstream of Wit. However, our data do not exclude the possibility that Imp acts downstream to communicate an anterograde cellular response to Wit signaling that modifies synaptic growth and plasticity. Synaptic development, homeostasis, and modulation of synaptic activities are important elements of functional neural circuits that depend on bi-directional signaling and communication between the synapse and cell body. While our observations underline the importance of Imp in synaptogenesis, we do not identify the target transcripts regulated by Imp. The composition of neuronal Imp RNP particles is an important area to pursue in future experiments. Indeed, there are many cytoplasmic RNA structures present within neurons and our knowledge of their functions is rudimentary [50,77]. Visual screens based on active cytoplasmic transport of RNP complexes should continue to provide an important tool for identifying additional gene products that regulate the transport and localization of mRNAs.

## Supporting Information

Figure S1 Chemical Disruption of Microtubules Blocks GFP-Imp Localization in the Oocyte GFP-Imp distribution was examined in egg chambers from females fed on colcemid-laced yeast paste for 24 hours. Disruption of microtubules blocks the accumulation of GFP-Imp seen in the wild type oocyte. GFP-Imp normally accumulates at the oocyte posterior by stage 10 (see Figure 2A'), but incubating the egg chambers in the actin inhibitor, cytochalasin D, causes Imp to disperse (posterior is at right side of image).
(1.6 MB TIF)

Figure S2 *Imp* Alleles and Transcripts (A) Schematic of the *Imp* genomic region indicates the site and orientation of the protein trap transposon (PTT) insertion; the intron/exon structure of the three *Imp* transgenes (*RE*, *SD*, and *KH*) used to overexpress Imp in select tissues; and the positions of the excision mutations *H44* and *H149*. In the *H44* mutation, the PTT element is both inverted and rearranged. A dashed line indicates the extent of the deletion caused by the *H149* mutation. The schematic is not drawn to scale.
(B) Western blot analysis of *Imp* alleles. Like the wild type control ovaries (lane 1), no GFP-Imp is detected in balanced excision lines *H44/+* (lane 2) or *H149/+* (lane 3). In ovaries from heterozygotes *H44/GFP-Imp* (lane 4) and *H149/GFP-Imp* (lane 5), as well as homozygous GFP-Imp/GFP-Imp ovaries (lane 6), the GFP-Imp product is detected by anti-GFP antibody. Probing a duplicate blot with anti-Imp antibody shows endogenous Imp resulting from expression of the wild type chromosomes (lanes 1–3, right panel), and GFP-tagged Imp from the PTT insertion chromosome (lanes 4–6, right panel); there is no evidence of truncated protein resulting from the *H44* or *H149* mutations. Each lane was loaded with equal total protein from ovary extracts.
(C) Flies overexpressing the *UAS-RE* transgene (*a-tubGAL4/+; UAS-RE/+*) during oogenesis produce an egg that is wild type in appearance. In contrast, flies overexpressing *UAS-SD* (*a-tubGAL4/+; UAS-SD/+*) produces an egg with fused, truncated dorsal appendages.
(2.4 MB TIF)

Figure S3 GFP-Imp Interacts with FMR1 and Motor Proteins (A) Immunoprecipitation from GFP-Imp ovary extracts. The precipitating antibodies are listed across the top of the figure, and the antibodies used to probe the western blot are listed along the side. Anti-GFP co-precipitates FMR1, and, reciprocally, anti-FMR1 pulls down GFP-Imp. Immunoprecipitation of the dynein intermediate chain (DIC) co-precipitates GFP-Imp. Minor amounts of kinesin also come down in these reactions. As a negative control, the reaction was carried out in the absence of antibodies. The first lane in the upper panel is derived from a shorter film exposure, which shows the GFP-Imp protein bands more clearly.
(B) FMR1, shown in red, immunolocalizes with a subset of the GFP-Imp particles (green) present in segmental axons. Co-localizing particles appear yellow. Bar, 5 μm. In the panel on the right, magnification is increased by a factor of two, providing better resolution of individual particles.
(4.9 MB TIF)

Video S1 GFP-tagged Ypsilon Schachtel in Nurse Cells GFP-tagged Ypsilon Schachtel particles move in a linear fashion within nurse cells. Acquisition was 1 fps for 3 minutes. Playback is 15 fps. Bar, 5 μm.
(4.1 MB MOV)

Video S2 GFP-tagged Squid in Nurse Cells GFP-tagged Squid particles move in a linear fashion within nurse cells. Acquisition was 1 fps for 3 minutes. Playback is 15 fps. Bar, 5 μm.
(5.7 MB MOV)

Video S3 GFP-Imp in Nurse Cells GFP-Imp particles move in a linear fashion within nurse cells. Acquisition was 1 fps for 3 minutes. Playback is 15 fps. Bar, 5 μm.
(6.3 MB MOV)

Video S4 GFP-Imp in Segmental Axons GFP-Imp particles exhibit rapid, bidirectional movement in segmental axons. Acquisition was .5 fps for 90 seconds. Playback is 15 fps. Bar, 5 μm.
(2.0 MB MOV)

Video S5 Larval Motility in *H44/H149* Transheterozygotes 20 s video; 30 fps.
(5.3 MB MOV)

Video S6 Larval Motility in Wild Type Sibling Heterozygous Larvae 10 s video; 30 fps.
(2.9 MB MOV)

Video S7 *H44/H149* Transheterozygous Mutant Flies Exhibit Severe Neuromuscular Dysfunction and Reduced Molitity 24 s video; 30 fps.
(7.3 MB MOV)

Video S8 WT Sibling Adult Progeny Exhibit Rapid, Unimpaired Motility 5 s video; 30 fps.
(1.3 MB MOV)

Video S9 Larval Motility Following Overexpression of Imp-RE in Neurons (*elav-GAL4/+; UAS-RE/+*
21 s video; 30 fps.
(6.0 MB MOV)

Video S10 Wild Type Sibling Larvae 10 s video; 30 fps.
(2.8 MB MOV)

Video S11 Overexpression of Imp-RE in Neurons (*elav-GAL4/+; UAS-RE/+)* Generates Severe Neuromuscular Dysfunction and Reduced Motility 18 s video; 30 fps.
(5.5 MB MOV)

Video S12 Wild type Sibling Flies Show Normal Motility 10 s video; 30 fps.
(2.9 MB MOV)

### Accession Numbers

The National Center for Biotechnology Information (NCBI) database (http://www.ncbi.nlm.nih.gov/gquery/gquery.fcgi?itool=toolbar) accession numbers for IGF-II mRNA-binding protein homologs are D. melanogaster isoforms NP_001036268, NP_727457, NP_727456, NP_727455, NP_727454, NP_727453, NP_727452, NP_727451, NP_511111; X. laevis, O57526 and O73932; Gallus gallus, O42254; Danio rerio, Q9PW80; Homo sapiens, Q9NZI8 and O00425; Mus musculus, O88477 and Q9CPN8; Rattus norvegicus, Q8CGX0; Caenorhabditis elegans, CAJ58502; and Saccharomyces cerevisiae, NP_009670.

## Abbreviations

ΔGl: truncated Glued
Dhc: dynein heavy chain
Khc: kinesin I heavy chain
NMJ: neuromuscular junction
RNP: ribonucleoprotein
ZBP: zipcode-binding protein
